# Supplementary material for: Sequential in vitro enzymatic N-glycoprotein modification reveals site-specific rates of glycoenzyme processing
Source: J Biol Chem. 2022 Sep 9;298(10):102474. doi: 10.1016/j.jbc.2022.102474 (PMC9530959; doi:10.1016/j.jbc.2022.102474)
Supplement: Supplementary Material [file mmc1.docx]

**S1: Protein sequences and purification.**

**S1a: Reporter protein sequences**

**Erythropoietin**

001 HHHHHHHHMS GLNDIFEAQK IEWHEMSKGE LFTGVVPILV ELDGDVNGHK

051 FSVRGEGEGD ATNGKLTLKF ICTTGKLPVP WPTLVTTLTY GVQCFSRYPD

101 HMKRHDFFKS AMPEGYVQER TISFKDDGTY KTRAEVKFEG DTLVNRIELK

151 GIDFKEDGNI LGHKLEYNFN SHNVYITADK QKNGIKANFK IRHNVEDGSV

201 QLADHYQQNT PIGDGPVLLP DNHYLSTQSV LSKDPNEKRD HMVLLEFVTA

251 AGITHGEFAS TSLYKKAGSE NLYFQGAPPR LICDSRVLER YLLEAKEAEN

301 ITTGCAEHCS LNENITVPDT KVNFYAWKRM EVGQQAVEVW QGLALLSEAV

351 LRGQALLVNS SQPWEPLQLH VDKAVSGLRS LTTLLRALGA QKEAISPPDA

401 ASAAPLRTIT ADTFRKLFRV YSNFLRGKLK LYTGEACRTG DR

**Etanercept**

001 HHHHHHHHMS GLNDIFEAQK IEWHEMSKGE ELFTGVVPIL VELDGDVNGH

051 KFSVRGEGEG DATNGKLTLK FICTTGKLPV PWPTLVTTLT YGVQCFSRYP

101 DHMKRHDFFK SAMPEGYVQE RTISFKDDGT YKTRAEVKFE GDTLVNRIEL

151 KGIDFKEDGN ILGHKLEYNF NSHNVYITAD KQKNGIKANF KIRHNVEDGS

201 VQLADHYQQN TPIGDGPVLL PDNHYLSTQS VLSKDPNEKR DHMVLLEFVT

251 AAGITHGEFA STSLYKKAGS ENLYFQGLPA QVAFTPYAPE PGSTCRLREY

301 YDQTAQMCCS KCSPGQHAKV FCTKTSDTVC DSCEDSTYTQ LWNWVPECLS

351 CGSRCSSDQV ETQACTREQN RICTCRPGWY CALSKQEGCR LCAPLRKCRP

401 GFGVARPGTE TSDVVCKPCA PGTFSNTTSS TDICRPHQIC NVVAIPGNAS

451 MDAVCTSTSP TRSMAPGAVH LPQPVSTRSQ HTQPTPEPST APSTSFLLPM

501 GPSPPAEGST GDEPKSCDKT HTCPPCPAPE LLGGPSVFLF PPKPKDTLMI

551 SRTPEVTCVV VDVSHEDPEV KFNWYVDGVE VHNAKTKPRE EQYNSTYRVV

601 SVLTVLHQDW LNGKEYKCKV SNKALPAPIE KTISKAKGQP REPQVYTLPP

651 SREEMTKNQV SLTCLVKGFY PSDIAVEWES NGQPENNYKT TPPVLDSDGS

701 FFLYSKLTVD KSRWQQGNVF SCSVMHEALH NHYTQKSLSL SPGK

**Protein disulfide isomerase**

001 MKFSAGAVLS WSSLLLASSV FAQQEAVAPE DSAVVKLATD SFNEYIQSHD

051 LVLAEFFAPW CGHCKNMAPE YVKAAETLVE KNITLAQIDC TENQDLCMEH

101 NIPGFPSLKI FKNSDVNNSI DYEGPRTAEA IVQFMIKQSQ PAVAVVADLP

151 AYLANETFVT PVIVQSGKID ADFNATFYSM ANKHFNDYDF VSAENADDDF

201 KLSIYLPSAM DEPVVYNGKK ADIADADVFE KWLQVEALPY FGEIDGSVFA

251 QYVESGLPLG YLFYNDEEEL EEYKPLFTEL AKKNRGLMNF VSIDARKFGR

301 HAGNLNMKEQ FPLFAIHDMT EDLKYGLPQL SEEAFDELSD KIVLESKAIE

351 SLVKDFLKGD ASPIVKSQEI FENQDSSVFQ LVGKNHDEIV NDPKKDVLVL

401 YYAPWCGHCK RLAPTYQELA DTYANATSDV LIAKLDHTEN DVRGVVIEGY

451 PTIVLYPGGK KSESVVYQGS RSLDSLFDFI KENGHFDVDG KALYEEAQEK

501 AAEEADADAE LADEEDAISG SGENLYFQGS SFLVQSGMSK GEELFTGVVP

551 ILVELDGDVN GHKFSVRGEG EGDATNGKLT LKFICTTGKL PVPWPTLVTT

601 LTYGVQCFSR YPDHMKRHDF FKSAMPEGYV QERTISFKDD GTYKTRAEVK

651 FEGDTLVNRI ELKGIDFKED GNILGHKLEY NFNSHNVYIT ADKQKNGIKA

701 NFKIRHNVED GSVQLADHYQ QNTPIGDGPV LLPDNHYLST QSVLSKDPNE

751 KRDHMVLLEF VTAAGITHGM SGLNDIFEAQ KIEWHEHHHH HHHH

**CD16a**

001 HHHHHHHHMS GLNDIFEAQK IEWHEMSKGE ELFTGVVPIL VELDGDVNGH

051 KFSVRGEGEG DATNGKLTLK FICTTGKLPV PWPTLVTTLT YGVQCFSRYP

101 DHMKRHDFFK SAMPEGYVQE RTISFKDDGT YKTRAEVKFE GDTLVNRIEL

151 KGIDFKEDGN ILGHKLEYNF NSHNVYITAD KQKNGIKANF KIRHNVEDGS

201 VQLADHYQQN TPIGDGPVLL PDNHYLSTQS VLSKDPNEKR DHMVLLEFVT

251 AAGITHGEFS SENLYFQGRT EDLPKAVVFL EPQWYRVLEK DSVTLKCQGA

301 YSPEDNSTQW FHNESLISSQ ASSYFIDAAT VDDSGEYRCQ TNLSTLSDPV

351 QLEVHIGWLL LQAPRWVFKE EDPIHLRCHS WKNTALHKVT YLQNGKGRKY

401 FHHNSDFYIP KATLKDSGSY FCRGLVGSKN VSSETVNITI TQG

**SARS-CoV-2 spike glycoprotein**

0001 MFVFLVLLPL VSSQCVNLTT RTQLPPAYTN SFTRGVYYPD KVFRSSVLHS

0051 TQDLFLPFFS NVTWFHAIHV SGTNGTKRFD NPVLPFNDGV YFASTEKSNI
0101 IRGWIFGTTL DSKTQSLLIV NNATNVVIKV CEFQFCNDPF LGVYYHKNNK

0151 SWMESEFRVY SSANNCTFEY VSQPFLMDLE GKQGNFKNLR EFVFKNIDGY

0201 FKIYSKHTPI NLVRDLPQGF SALEPLVDLP IGINITRFQT LLALHRSYLT

0251 PGDSSSGWTA GAAAYYVGYL QPRTFLLKYN ENGTITDAVD CALDPLSETK

0301 CTLKSFTVEK GIYQTSNFRV QPTESIVRFP NITNLCPFGE VFNATRFASV

0351 YAWNRKRISN CVADYSVLYN SASFSTFKCY GVSPTKLNDL CFTNVYADSF

0401 VIRGDEVRQI APGQTGKIAD YNYKLPDDFT GCVIAWNSNN LDSKVGGNYN

0451 YLYRLFRKSN LKPFERDIST EIYQAGSTPC NGVEGFNCYF PLQSYGFQPT

0501 NGVGYQPYRV VVLSFELLHA PATVCGPKKS TNLVKNKCVN FNFNGLTGTG

0551 VLTESNKKFL PFQQFGRDIA DTTDAVRDPQ TLEILDITPC SFGGVSVITP

0601 GTNTSNQVAV LYQDVNCTEV PVAIHADQLT PTWRVYSTGS NVFQTRAGCL

0651 IGAEHVNNSY ECDIPIGAGI CASYQTQTNS PGGSGSVASQ SIIAYTMSLG

0701 AENSVAYSNN SIAIPTNFTI SVTTEILPVS MTKTSVDCTM YICGDSTECS

0751 NLLLQYGSFC TQLNRALTGI AVEQDKNTQE VFAQVKQIYK TPPIKDFGGF

0801 NFSQILPDPS KPSKRSFIED LLFNKVTLAD AGFIKQYGDC LGDIAARDLI

0851 CAQKFNGLTV LPPLLTDEMI AQYTSALLAG TITSGWTFGA GAALQIPFAM

0901 QMAYRFNGIG VTQNVLYENQ KLIANQFNSA IGKIQDSLSS TASALGKLQD

0951 VVNQNAQALN TLVKQLSSNF GAISSVLNDI LSRLDPPEAE VQIDRLITGR

1001 LQSLQTYVTQ QLIRAAEIRA SANLAATKMS ECVLGQSKRV DFCGKGYHLM

1051 SFPQSAPHGV VFLHVTYVPA QEKNFTTAPA ICHDGKAHFP REGVFVSNGT

1101 HWFVTQRNFY EPQIITTDNT FVSGNCDVVI GIVNNTVYDP LQPELDSFKE

1151 ELDKYFKNHT SPDVDLGDIS GINASVVNIQ KEIDRLNEVA KNLNESLIDL

1201 QELGKYEQGS GGYIPEAPRD GQAYVRKDGE WVLLSTFLGG SHHHHHH

**S1b: CD16a purification**

**S1c: PDI purification**

**S1d: Etanercept purification**

**S1e: Spike purification**

**S1f: Enzyme purification**

**S1g: Linear representation of reporter proteins**

**S2: SARS-CoV-2 spike glycoprotein site occupancy**

**S3: Site-specific N-glycan processing rates of SARS-CoV-2 spike glycoprotein**

**S4: Levels of GlcNAc3Man4 intermediate during MAN2A1 processing**

**S5: Site occupancy of CD16a Sequon 1 (N056) expressed in HEK293F cells.**

**S6: Site occupancy of CD16a Sequon 2 (N063) expressed in HEK293F cells.**

**S7: Site occupancy of CD16a Sequon 3 (N092) expressed in HEK293F cells.**

**S8: Site occupancy of CD16a Sequon 4 (N180) expressed in HEK293F cells.**

**S9: Site occupancy of CD16a Sequon 5 (N187) expressed in HEK293F cells.**

**S10: Site occupancy of PDI Sequon 1 (N082) expressed in HEK293F cells**


**S11: Site occupancy of PDI Sequon 2 (N117) expressed in HEK293F cells**

**S12: Site occupancy of PDI Sequon 3 (N155) expressed in HEK293F cells**

**S13: Site occupancy of PDI Sequon 4 (N174) expressed in HEK293F cells**

**S14: Site occupancy of PDI Sequon 5 (N425) expressed in HEK293F cells**

**S15: Site occupancy of etanercept sequon 1 (N149) expressed in HEK293F cells**

**S16a: Site occupancy of etanercept sequon 2 (N171) expressed in HEK293F cells**

**S16b: Representative MS2s of hybrid structure from Lec1-expressed Etanercept sequon 2 (N171)**

**
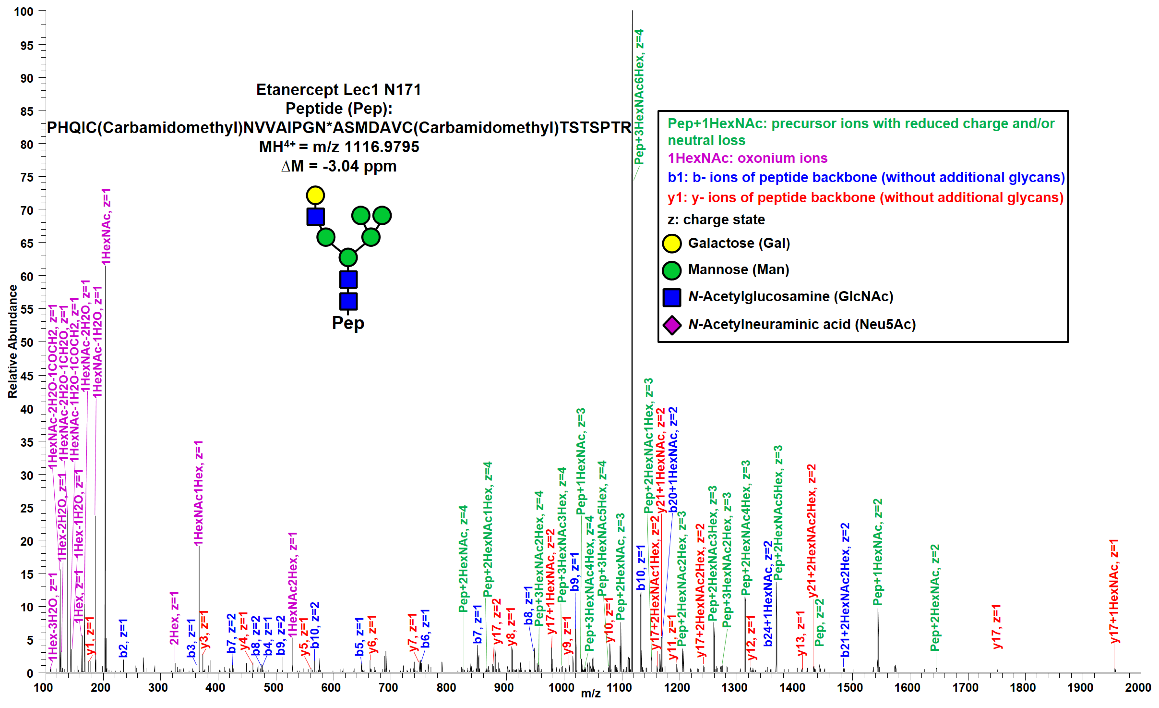
**


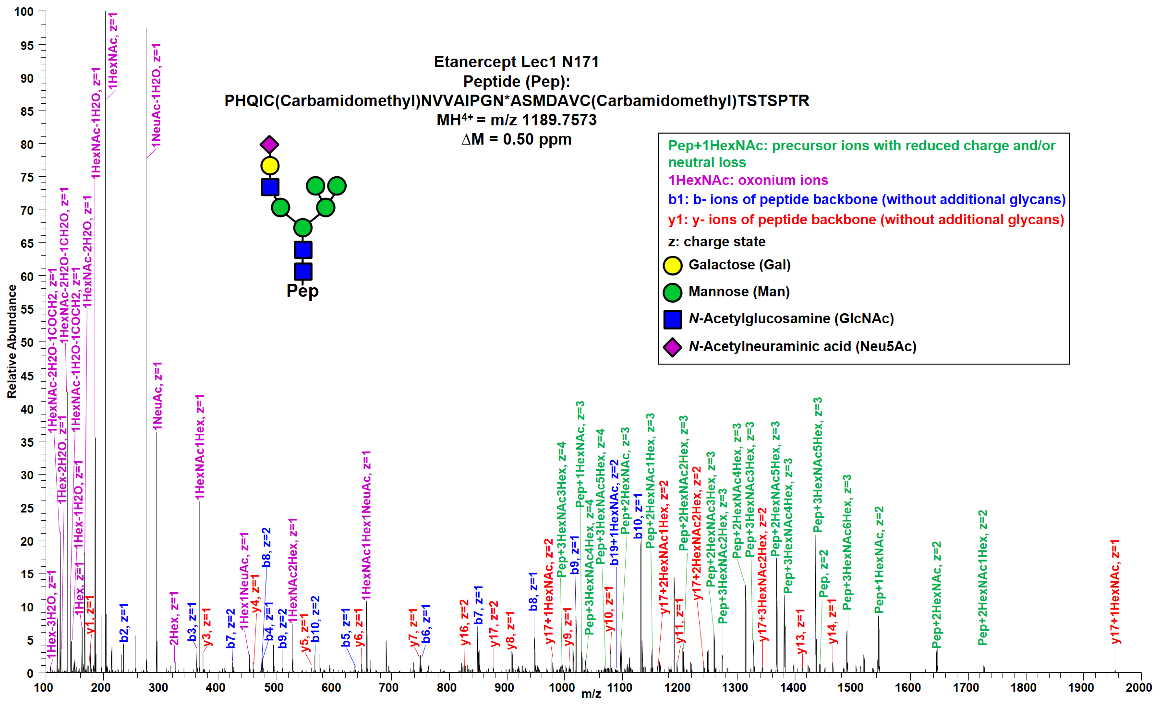


**S17: Site occupancy of etanercept sequon 3 (N317) expressed in HEK293F cells**

**S18: Site occupancy of erythropoietin sequon 1 (N051) expressed in HEK293F cells**

**S19: Site occupancy of erythropoietin sequon 2 (N065) expressed in HEK293F cells**

**S20: Site occupancy of erythropoietin sequon 3 (N110) expressed in HEK293F cells**


**S21: Site occupancy of SARS-CoV-2 spike glycoprotein sequon 1 (N0017) expressed in HEK293F cells**

**S22: Site occupancy of SARS-CoV-2 spike glycoprotein sequon 2 (N0061) expressed in HEK293F cells**

**S23: Site occupancy of SARS-CoV-2 spike glycoprotein sequon 3 (N0074) expressed in HEK293F cells**

**S24: Site occupancy of SARS-CoV-2 spike glycoprotein sequon 4 (N0122) expressed in HEK293F cells**

**S25: Site occupancy of SARS-CoV-2 spike glycoprotein sequon 5 (N0149) expressed in HEK293F cells**

**S26: Site occupancy of SARS-CoV-2 spike glycoprotein sequon 6 (N0165) expressed in HEK293F cells**

**S27: Site occupancy of SARS-CoV-2 spike glycoprotein sequon 7 (N0234) expressed in HEK293F cells**

**S28: Site occupancy of SARS-CoV-2 spike glycoprotein sequon 8 (N0282) expressed in HEK293F cells**

**S29: Site occupancy of SARS-CoV-2 spike glycoprotein sequon 9 (N0331) expressed in HEK293F cells**

**S30: Site occupancy of SARS-CoV-2 spike glycoprotein sequon 10 (N0343) expressed in HEK293F cells**

**S31: Site occupancy of SARS-CoV-2 spike glycoprotein sequon 11 (N0603) expressed in HEK293F cells**

**S32: Site occupancy of SARS-CoV-2 spike glycoprotein sequon 12 (N0616) expressed in HEK293F cells**

**S33: Site occupancy of SARS-CoV-2 spike glycoprotein sequon 13 (N0657) expressed in HEK293F cells**

**S34: Site occupancy of SARS-CoV-2 spike glycoprotein sequon 14 (N0709) expressed in HEK293F cells**

**S35: Site occupancy of SARS-CoV-2 spike glycoprotein sequon 15 (N0717) expressed in HEK293F cells**

**S36: Site occupancy of SARS-CoV-2 spike glycoprotein sequon 16 (N0801) expressed in HEK293F cells**

**S37: Site occupancy of SARS-CoV-2 spike glycoprotein sequon 17 (N1074) expressed in HEK293F cells**

**S38: Site occupancy of SARS-CoV-2 spike glycoprotein sequon 18 (N1098) expressed in HEK293F cells**

**S39: Site occupancy of SARS-CoV-2 spike glycoprotein sequon 19 (N1134) expressed in HEK293F cells**

**S40: Site occupancy of SARS-CoV-2 spike glycoprotein sequon 20 (N1158) expressed in HEK293F cells**

**S41: Site occupancy of SARS-CoV-2 spike glycoprotein sequon 21 (N1173) expressed in HEK293F cells**

**S42: Site occupancy of SARS-CoV-2 spike glycoprotein sequon 22 (N1194) expressed in HEK293F cells**

**S43: Example MS1 total ion chromatogram for PDI-N425 (Sequon 5) MGAT1 timecourse reaction**
