# Supplementary material for: Sequential in vitro enzymatic N-glycoprotein modification reveals site-specific rates of glycoenzyme processing
Source: J Biol Chem. 2022 Sep 9;298(10):102474. doi: 10.1016/j.jbc.2022.102474 (PMC9530959; doi:10.1016/j.jbc.2022.102474)
Supplement: Supporting Tables and Figures [file mmc2.docx]

**Supporting Information**

**S1. Protein sequences, purification, and linear representation.**

**S2. SARS-CoV-2 spike glycoprotein site occupancy
S3. Site-specific N-glycan processing rates of SARS-CoV-2 spike glycoprotein**

**S4. Levels of GlcNAc3Man4 intermediate during MAN2A1 processing**

**S5-9. N-glycan site occupancy on CD16a when expressed in WT-HEK293F and Lec1-HEK293F cells.**

**S10-14. N-glycan site occupancy on PDI when expressed in Lec1-HEK293F cells.**

**S15-17. N-glycan site occupancy on etanercept when expressed in WT-HEK293F and Lec1-HEK293F cells including S16B showing fragmentation of hybrid structures on Sequon 2 (N171) from Lec1-HEK293F cells**

**S18-20. N-glycan site occupancy on erythropoietin when expressed in WT-HEK293F and Lec1-HEK293F cells.**

**S21-42. N-glycan site occupancy on SARS-CoV-2 spike glycoprotein when expressed in WT-HEK293F and Lec1-HEK293F cells.**

**S43: Example MS1 total ion chromatogram for PDI-N425 (Sequon 5) MGAT1 timecourse reaction**

**Table S1. Glycan occupancy at each site of protein disulfide-isomerase (PDI1), etanercept, erythropoietin (EPO), low affinity immunoglobulin gamma Fc region receptor III-A (FCGR3A/etanercept), and spike glycoprotein (S) from wild type and Lec1 (GnT1-/-) HEK293 cells.**

**Table S2. Glycan types identified at each site of protein disulfide-isomerase (PDI1), etanercept, erythropoietin (EPO), low affinity immunoglobulin gamma Fc region receptor III-A (FCGR3A/etanercept), and spike glycoprotein (S) from wild type and Lec1 (GnT1-/-) HEK293 cells.**

**Table S3: Glycan topologies identified at each site of protein disulfide-isomerase (PDI1), etanercept, erythropoietin (EPO), low affinity immunoglobulin gamma Fc region receptor III-A (FCGR3A/etanercept), and spike glycoprotein (S) from wild type and Lec1 (GnT1-/-) HEK293 cells.** Asn(N)# indicates the numbers of asparagines in protein sequences. In toplogies: N=HexNAc, H=Hexose (Hex), F=Fucose (Fuc), A=Neu5Ac. In fucosylation: NoFuc=No Fuc identified; 1Core=One Fuc identified at core position; 1Term=One Fuc identified at terminal position; 1Core and 1Term=One Fuc identified as a mixture of core and terminal positions; 1Core1Term=Two Fuc identified and one is at core and the other is at terminal; 2Term=Two Fuc identified at terminal positions; 1Core1Term and 2Term=Two Fuc identified as a mixture of core and terminal positions; 1Core2Term=Three Fuc identified and one is at core and the others are at terminal; 3Term=Three Fuc identified at terminal positions; 1Core2Term and 3Term=Three Fuc identified as a mixture of core and terminal positions; 1Core3Term=Four Fuc identified and one is at core and the others are at terminal; 4Term=Four Fuc identified at terminal positions; 1Core3Term and 4Term=Four Fuc identified as a mixture of core and terminal positions; 1Core4Term=Five Fuc identified and one is at core and the others are at terminal; 5Term=Five Fuc identified at terminal positions; 1Core4Term and 5Term=Five Fuc identified as a mixture of core and terminal positions; 1Core5Term=Six Fuc identified and one is at core and the others are at terminal; 6Term=Six Fuc identified at terminal positions; 1Core5Term and 6Term=Six Fuc identified as a mixture of core and terminal positions.
